# Clinical efficacy and IL-17 targeting mechanism of *Indigo naturalis* as a topical agent in moderate psoriasis

**DOI:** 10.1186/s12906-017-1947-1

**Published:** 2017-09-02

**Authors:** Hui-Man Cheng, Yang-Chang Wu, Qingmin Wang, Michael Song, Jackson Wu, Dion Chen, Katherine Li, Eric Wadman, Shung-Te Kao, Tsai-Chung Li, Francisco Leon, Karen Hayden, Carrie Brodmerkel, C. Chris Huang

**Affiliations:** 1 Department of Integration of Traditional Chinese and Western Medicine, China Medical University Hospital, Taichung, Taiwan; 2 School of Chinese Medicine, College of Chinese Medicine, China Medical University, Taichung, Taiwan; 3 Chinese Medicine Research and Development Center, China Medical University Hospital, Taichung, Taiwan; 4 Graduate Institute of Natural Products, College of Pharmacy, Kaohsiung Medical University, Kaohsiung, Taiwan; 5 Janssen Research & Development, LLC, Spring House, PA USA; 6 College of Pharmacy, Taipei Medical University, Taipei, Taiwan; 7 Department of Internal Chinese Medicine, China Medical University Hospital, Taichung, Taiwan; 8 Department of Public Health, College of Public Health, China Medical University, Taichung, Taiwan

**Keywords:** *Indigo naturalis*, Gene expression, Mechanism of action, Psoriasis

## Abstract

**Background:**

*Indigo naturalis* is a Traditional Chinese Medicine (TCM) ingredient long-recognized as a therapy for several inflammatory conditions, including psoriasis. However, its mechanism is unknown due to lack of knowledge about the responsible chemical entity. We took a different approach to this challenge by investigating the molecular profile of *Indigo naturalis* treatment and impacted pathways.

**Methods:**

A randomized, double-blind, placebo-controlled clinical study was conducted using *Indigo naturalis* as topical monotherapy to treat moderate plaque psoriasis in a Chinese cohort (*n* = 24). Patients were treated with *Indigo naturalis* ointment (*n* = 16) or matched placebo (*n* = 8) twice daily for 8 weeks, with 1 week of follow-up.

**Results:**

At week 8, significant improvements in Psoriasis Area and Severity Index (PASI) scores from baseline were observed in *Indigo naturalis*-treated patients (56.3% had 75% improvement [PASI 75] response) compared with placebo (0.0%). A gene expression signature of moderate psoriasis was established from baseline skin biopsies, which included the up-regulation of the interleukin (IL)-17 pathway as a key component; *Indigo naturalis* treatment resulted in most of these signature genes returning toward normal, including down-regulation of the IL-17 pathway. Using an in vitro keratinocyte assay, an IL-17-inhibitory effect was observed for tryptanthrin, a component of *Indigo naturalis*.

**Conclusions:**

This study demonstrated the clinical efficacy of *Indigo naturalis* in moderate psoriasis, and exemplified a novel experimental medicine approach to understand TCM targeting mechanisms.

**Trial registration:**

NCT01901705.

**Electronic supplementary material:**

The online version of this article (10.1186/s12906-017-1947-1) contains supplementary material, which is available to authorized users.

## Background

Psoriasis is a chronic inflammatory skin disease afflicting 1–3% of the world’s population [1] and 0.12–0.47% of the Chinese population [2–4]. Psoriasis is characterized by hyperproliferation of keratinocytes and dermal infiltration by activated T cells, neutrophils, and dendritic cells. The infiltrated psoriatic lesions have significantly increased levels of the CD4 T-cell subsets T helper (Th)1 and Th17 [5]. Clinical studies have shown that neutralizing tumor necrosis factor-alpha (TNF-α), interleukin (IL)-12/23, or IL-17A is efficacious, demonstrating the central role of Th1/Th17 cells in psoriasis pathogenesis [6–12]. Several classes of therapy are available to treat patients with psoriasis including topical agents (e.g., corticosteroids, vitamin D_3_, salicylic acid, retinoids) and systemic agents (e.g., methotrexate, acitretin, cyclosporine, biologics). Topicals are regularly used as first-line options to treat mild-to-moderate psoriasis; however, long-term use of topical agents, such as corticosteroids, has been associated with adverse events such as atrophy and striae formation [13]. Despite the availability of these treatments, topical Traditional Chinese Medicines (TCM), with *Indigo naturalis* as a key component, are commonly used as alternate therapies, especially in the Asia Pacific region [14–16].

*Indigo naturalis*, a Chinese herb known as Qing Dai, is a dried pigment prepared from several plant species including *Baphicacanthus cusia*. Recently, clinical studies have demonstrated that *Indigo naturalis* used as topical monotherapy is efficacious in treating patients with mild-to-moderate psoriasis [17–22], although the validity of these studies is challenged by intra-patient treatment comparison designs [23]. One component of *Indigo naturalis*, indirubin, was reported to inhibit cyclin-dependent kinase [24, 25] and signal transducer and activator of transcription-3 (STAT3) activities [26], and keratinocyte proliferation in vitro [17, 20, 27]. Another compound, tryptanthrin, was reported to inhibit interferon-γ production by lymphocytes from Peyer’s Patches [28], and nitric oxide and prostaglandin E2 synthesis by murine macrophages [29]. Moreover, *Indigo naturalis* extract has been shown to inhibit oxygen generation and elastase release in formyl-methionyl-leucyl-phenylalanine-induced human neutrophils in vitro [21]. Still, as with most TCM, the therapeutically- active chemical entities of *Indigo naturalis* are poorly defined. Other plant extracts have also been investigated as topical agents for the treatment of psoriasis in controlled clinical trials [30], including a topical cream with *mahonia* extracts (*Mahonia aquifolium*), in which the potential active chemical ingredient was speculated to have an anti-inflammatory mechanism [31].

We report the first randomized, double-blind, placebo-controlled clinical trial to evaluate the pharmacological effect of *Indigo naturalis* as a single topical agent (topical ointment) in Chinese patients with moderate plaque-type psoriasis. Additionally, global gene expression analysis of affected skin was conducted to establish a molecular signature of moderate psoriasis (in our Chinese cohort and in a predominantly White cohort [32]), and used to assess the impact of *Indigo naturalis* treatment. Finally, major chemical components of *Indigo naturalis* were evaluated for anti-IL-17-induced cytokine release in cultured keratinocytes.

## Methods

### Clinical study

This randomized, double-blinded, placebo-controlled study was registered with the Institutional Review Board of China Medical University Hospital, Taiwan on December 13, 2012. All patients provided written informed consent prior to enrollment. Patients were eligible to be included in the study if at baseline they were 20–65 years, had a diagnosis of plaque-type psoriasis for ≥6 months, a Physician's Global Assessment (PGA) score of 2–3, <20% total body surface area (BSA) involvement, and a target plaque of ≥4 cm^2^.

Patients were excluded if they had non-plaque psoriasis, rebound/flare of chronic psoriasis, history of psoriatic arthritis, current drug-induced psoriasis, were pregnant/nursing/planning pregnancy (men and women), used biologics within 3 months or 5 times the half-life, received phototherapy/systemic treatment within 4 weeks, topicals within 2 weeks, any systemic immunosuppressants within 4 weeks, lithium/antimalarial/intramuscular gold within 4 weeks, tested positive for HIV/hepatitis B/C, had a history of alcohol/drug abuse, clinically-significant laboratory abnormality, sensitivity to Chinese herbs/olive oil/microcrystalline wax/Vaseline, had current signs/symptoms of severe, progressive, or uncontrolled medical conditions, or were participating concurrently in an investigational study. Patients were asked to maintain a daily diary to document treatment compliance and adverse events.

### Study agents

**Fig. 1:**
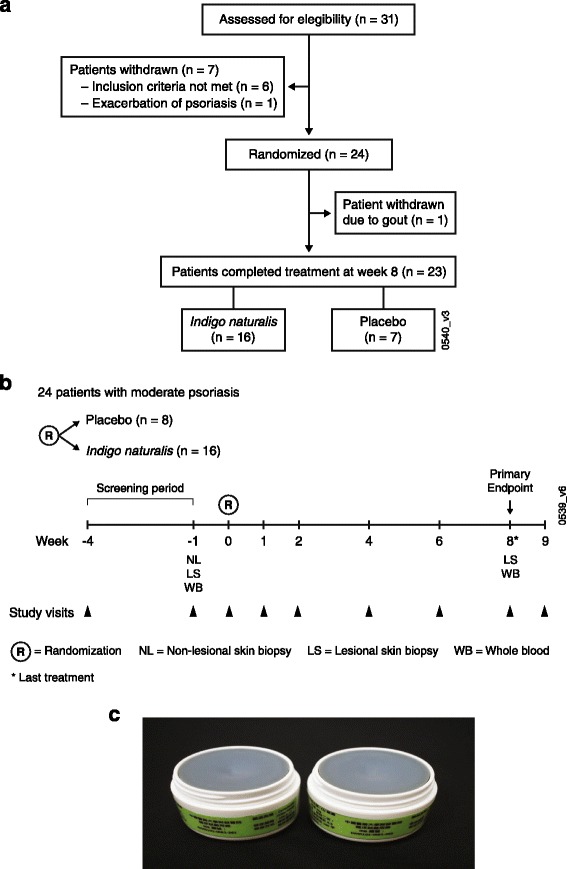
Study design. (**a**) Patient disposition; (**b**) study design; (**c**) study agent and placebo. The *Indigo naturalis* ointment used was a mixture (1:10) of *Indigo naturalis* powder and vehicle (Vaseline, microcrystalline wax, and olive oil [5:6:9]). Placebo preparation used was a mixture of blue dye powder (Indigo carmine aluminum lake [Blue #2] and Allura red AC aluminum lake [Red #40]) and vehicle

*Indigo naturalis* (Qing Dai) was extracted from the aerial part of *Baphicacanthus cusia* and stored as dried powder*.* The *Indigo naturalis* ointment was composed of a 1:10 mixture of *Indigo naturalis* powder and a vehicle consisting of Vaseline: microcrystalline wax: olive oil (5:6:9 ratio) (Fig. 1c). The placebo was a mixture of blue dye powder (54.8% Indigo carmine aluminum lake [Blue #2] and 45.2% Allura Red AC aluminum lake [Red #40] powders), Vaseline, microcrystalline wax, and olive oil. Across three batches of extracts, consistent indigo (2.83%) and indirubin (0.24%) concentration levels were maintained. To maintain the blind, both the *Indigo naturalis* and the placebo-control agents had a blue color (Fig. 1c). The blue dye of *Indigo naturalis* is not considered an active ingredient. Both study agents were manufactured at the same Good Manufacturing Practice facility (Sheng Chang Pharmaceutical Co., Ltd., Taoyuan, Taiwan). High-performance liquid chromatography was used for quality control of the final product.

### Sample size

Sample size was calculated using the hypothesis that a decrease in Psoriasis Area and Severity Index (PASI) scores at week 8 would be observed following *Indigo naturalis* treatment. It has been reported that a sample size of 7 placebo patients and 12 *Indigo naturalis* patients was needed to achieve a statistical power of 90% at 0.05 level of significance [19]. Assuming a patient follow-up rate of 0.9, it was determined that at least 8 placebo patients and 16 *Indigo naturalis* patients would be needed to reach statistical significance.

### Study design

A total of 24 patients with moderate psoriasis were randomized 1:2 at week 0 to placebo (*n* = 8) or *Indigo naturalis* (*n* = 16) (Fig. 1a). Prior to week 8, one patient (placebo) discontinued due to gout. Patients were given and instructed to use the topical ointment twice daily for 8 weeks or until complete skin clearance was achieved, whichever occurred first. The amount of ointment used was assessed by estimating the surface area of each psoriatic lesion. To calculate the number of fingertip units (FTUs) to be used, 1 FTU = 0.5 g = 100 cm^2^ of surface area. In the 4-week screening period prior to treatment, whole blood, lesional, and nonlesional biopsy samples were collected. At week 0, patients received their first treatment. Subsequent visits occurred at weeks 1, 2, 4, 6, 8, and 9. Final whole blood and lesional samples were collected at week 8 (Fig. 1b).

### Study assessments

Safety and tolerability were evaluated through week 9. Safety evaluations included adverse event assessments, physical exams, electrocardiogram measurements, and hematology and blood chemistry analyses. Tolerability was evaluated through week 9 using: PASI to measure the severity of psoriasis ranging from a score of 0 (no psoriasis) to 72 (very severe); BSA involvement with psoriasis; PGA scores ranging from 0 to 5: cleared (0), minimal (1), mild (2), moderate (3), marked (4), and severe (5); and Overall Target Plaque Severity Scores (OTPSS) that measures plaque severity ranging from a score of 0 (no evidence of disease) to 8 (very severe).

### Skin biopsy and gene expression microarray

Two adjacent 4-mm punch biopsy specimens of lesional and nonlesional skin were obtained from each patient at week 0. A repeat biopsy was taken from the same lesional area 8 weeks after treatment with *Indigo naturalis* or placebo. Ribonucleic acid (RNA) was amplified using the NuGen Ovation® RNA Amplification System (Nugen, San Carlos, CA). The fragmented cDNA was hybridized to Affymetrix HT HG-U133 + PM Array (Affymetrix, Santa Clara, CA).

Microarray data were analyzed using ArrayStudio (Omicsoft, Cary, NC). Overall, 68 samples passed quality control analysis including 23 nonlesional samples and 24 lesional samples at baseline, and 21 lesional samples at week 8. Group comparisons were assessed using a general linear model (fold-change cutoff = 1.5) followed by multiple test correction using false discovery rate methodology (cutoff = 0.05). Pathway level analysis of differentially expressed genes (DEG) was conducted using enrichment analysis in Ingenuity Pathway Analysis (http://www.ingenuity.com).

### Inhibition of IL-17-induced cytokine release in keratinocytes

Normal human adult keratinocytes (Lonza) were seeded into 48-well culture plates and cultured to passage three with an approximate density of 70–80%. Keratinocytes were stimulated with IL-17A (100 ng/mL) (R&D Systems, Inc., Minneapolis, MN) and treated with varying concentrations of isatin, tryptanthrin, and indirubin (Sigma-Aldrich, St. Louis, MO), and a control compound, PD 0325901 [33]. PD 0325901 is a mitogen-activated protein kinase kinase (MEK) inhibitor; a similar compound has been demonstrated to inhibit IL-17-induced cytokine release [34]. Cell viability was confirmed using the PrestoBlue® assay (Thermo Fisher Scientific, Waltham, MA). Tryptanthrin showed no impact on cell viability at all testing concentrations. Tissue culture supernatants were analyzed for pro-inflammatory cytokines IL-6 and IL-8 using MesoScale Discovery V-plex assay (MesoScale Discovery, Rockville, MD). Data were analyzed using Graphpad Prism v5.0 (http://www.graphpad.com).

### Statistical analysis

Descriptive statistics such as mean, median, standard deviation, and proportion were used. No imputation of missing observations was performed. Safety was assessed by summarizing the incidence and type of adverse events through week 9. Bivariate statistical analysis such as the Chi-square test or Fisher’s exact test for categorical variables and two-sample t test for continuous variables were employed to evaluate the differences in baseline demographic and outcomes variables between active treatment and placebo groups.

## Results

### Baseline demographics and disease characteristics

Of the 31 patients screened, 24 patients were randomized 2:1 to receive *Indigo naturalis* (*n* = 16) or placebo (*n* = 8); 23 patients completed the study (Fig. 1a). All patients were Asian, and 70.8% were men (Table 1). At baseline, the extent of BSA involvement with psoriasis was approximately 9%; the mean PASI score was approximately 10; and the mean PGA score was approximately 3 (Table 1), consistent with moderate psoriasis [35].

**Table 1:** Baseline demographics and disease characteristics

	Placebo	*Indigo naturalis*	Total
Patients randomized, n	8	16	24
Men, n (%)	7 (87.5)	10 (62.5)	17 (70.8)
Race, n (%)			
Asian	8 (100)	16 (100)	24 (100)
Age, years	40.1 ± 10.9	39.3 ± 10.1	39.6 ± 10.1
Weight, kg	74.1 ± 16.7	73.2 ± 16.0	73.5 ± 15.9
Body mass index (kg/m^2^)	25.2 ± 4.6	25.9 ± 3.9	25.7 ± 4.0
Duration of psoriasis, years	14.9 ± 12.1	13.1 ± 11.1	13.7 ± 11.2
Age at diagnosis, years	25.3 ± 13.3	26.2 ± 12.0	25.7 ± 11.2
BSA involvement (%),	9.6 ± 5.8	8.4 ± 5.8	8.8 ± 5.7
PASI score (0–72)	11.1 ± 3.7	10.1 ± 4.3	10.4 ± 4.0
≥ 10, n (%)	4 (50.0)	8 (50.0)	12 (50.0)
< 10, n (%)	4 (50.0)	8 (50.0)	12 (50.0)
PGA score	3.3 ± 0.5	3.0 ± 0.5	3.1 ± 0.5
OTPSS	3.8 ± 0.5	3.6 ± 0.5	3.6 ± 0.5

Values are mean ± SD, unless otherwise indicated

*BSA* body surface area, *OTPSS* Overall Target Plaque Severity Score, *PASI* Psoriasis Area and Severity Index, *PGA* Physician's Global Assessment, *SD* standard deviation

### Efficacy

**Fig. 2:**
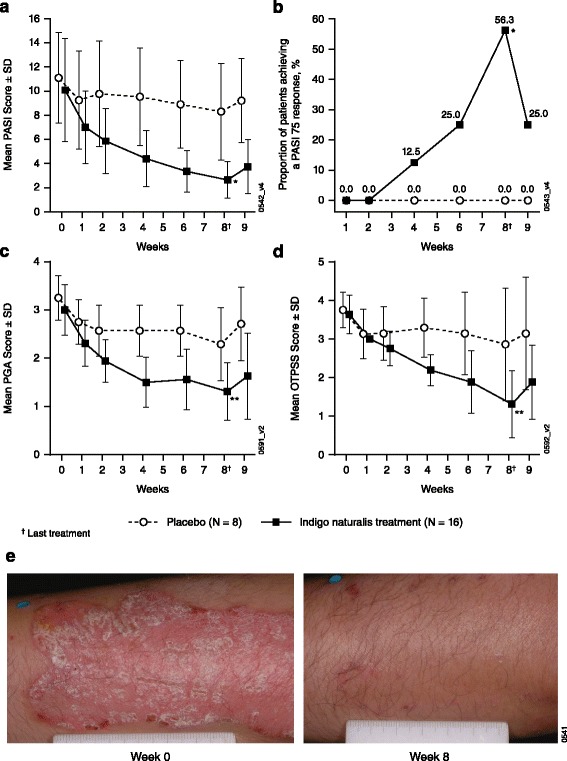
Efficacy assessments. (**a**) Mean PASI scores; (**b**) proportion of placebo- and *Indigo naturalis*-treated patients achieving a PASI 75 response (PASI 75 responders); (**c**) mean PGA scores; (**d**) and mean OTPSS in placebo- and *Indigo naturalis*-treated patients by study visit; (**e**) photo of a patient prior to *Indigo naturalis* treatment at week 0 (baseline) (*left panel*) and at week 8 following *Indigo naturalis* treatment (right panel). ^*^ *p* < 0.05 and ^**^ *p* < 0.005 vs. placebo. OTPSS, Overall Target Plaque Severity Score; PASI, Psoriasis Area and Severity Index; PGA, Physician's Global Assessmen t; SD, standard deviation

PASI scores were measured at baseline and each visit through week 9. Baseline PASI scores were similar between the two groups (Table 1). As early as week 2, *Indigo naturalis*-treated patients showed a statistically significant difference in mean PASI score compared with placebo-treated patients (*p* = 0.02; Fig. 2a). At week 8, the time of the last *Indigo naturalis* treatment, the mean PASI score of *Indigo naturalis* patients was 2.64 ± 1.5 representing a significant improvement from baseline (*p* = 0.01) and a significant difference compared with placebo patients (8.30 ± 4.0; *p* = 0.01; Fig. 2a). Moreover, 56.3% of *Indigo naturalis* patients achieved at least a 75% improvement in PASI (PASI 75 response) at week 8 compared with none of the placebo patients (0/7; 0.0%; *p* = 0.02; Fig. 2b). Consistent with PASI results, baseline PGA scores were similar between the two groups; however, a significant difference in mean PGA score was observed between *Indigo naturalis* patients (1.31 ± 0.6) and placebo patients at week 8 (2.29 ± 0.8; *p* < 0.003; Fig. 2c). Similarly, a significant difference in mean was observed between *Indigo naturalis* (1.31 ± 0.9) and placebo patients (2.86 ± 1.5) at week 8 (*p* = 0.003; Fig. 2d). An example of a skin lesion pre- and post-*Indigo naturalis* treatment is shown in Fig. 2e. At week 9 however, the mean PASI score for the *Indigo naturalis* patients was 3.74 ± 2.3, indicating diminished efficacy following termination of treatment.

### Safety

*Indigo naturalis* was well-tolerated. With an average duration of follow-up of approximately 9 weeks, the proportion of patients with at least one adverse event was comparable between treatment groups (placebo: 50.0%; *Indigo naturalis*: 44.0%; Table 2). The most common adverse event in the *Indigo naturalis* group was pruritus (25.0%), followed by rash and nasopharyngitis (each 12.5%) and single events of pruritus, gout, allergies, and pyrexia were reported in the placebo group (all 12.5%). One placebo-treated patient discontinued treatment prior to study completion (at week 3) due to an adverse event of gout (Fig. 1a).

**Table 2:** Patient safety data through week 9

	Placebo	*Indigo naturalis*
Patients treated, n	8	16
Patients with >1 adverse event, n (%)	4 (50.0)	7 (44.0)
Average duration of follow-up (weeks)	9.14	9.13
Average exposure (weeks)	8.16	8.13
Common adverse events, n (%)		
Pruritus	1 (12.5)	4 (25.0)
Rash	0 (0.0)	2 (12.5)
Nasopharyngitis	0 (0.0)	2 (12.5)
Abdominal distension	0 (0.0)	1 (6.3)
Constipation	0 (0.0)	1 (6.3)
Cough	0 (0.0)	1 (6.3)
Dizziness	0 (0.0)	1 (6.3)
Oropharyngeal pain	0 (0.0)	1 (6.3)
Gout	1 (12.5)	0 (0.0)
Allergies	1 (12.5)	0 (0.0)
Pyrexia	1 (12.5)	0 (0.0)

### Gene expression signature of moderate psoriasis

**Fig. 3:**
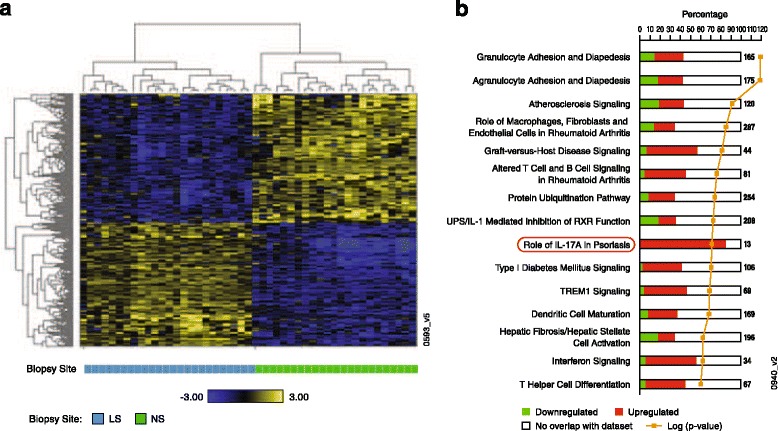
Moderate psoriasis signature. (**a**) Hierarchical clustering diagram of a moderate psoriasis signature generated from biopsies taken from lesional skin (LS; *blue bar*) and nonlesional skin (NS; *green bar* at the bottom) of the same patients at baseline; genes are represented as rows and samples as columns. Upregulated genes are shown in *yellow* and downregulated in *blue*; (**b**) enrichment of ingenuity pathways by moderate psoriasis gene signature. The stacked bar chart displays the number of up-regulated (*red*) and down-regulated (*green*) genes in each Ingenuity Canonical Pathway. The pathways are ranked by the *p*-value of a Fisher exact test from top to bottom (*orange line*; *p*-value listed in Additional file S1)

To gain a comprehensive understanding of the molecular characteristics of moderate psoriasis represented by this cohort, a global gene expression profile was generated from skin biopsies. Baseline lesional skin samples were compared with nonlesional samples from the same patients (*n* = 24). A total of 6845 probe sets with significant modulation (3384 up and 3461 down) were identified. The corresponding 4320 DEG define a disease signature of moderate psoriasis. A hierarchical clustering analysis showed distinct expression patterns of these genes in psoriatic lesions compared with nonlesional skin (Fig. 3a). To gain functional insight to the disease signature, a pathway enrichment analysis was conducted that resulted in 193 enriched pathways (see Additional file 1). The top 15 pathways (Fig. 3b) included the “Role of IL-17A in psoriasis,” indicating that the IL-17 pathway is a key component of moderate psoriasis.

Because our study population was composed entirely of Asian patients, we performed a comparison to another psoriasis population [32] that was predominantly White. That study population [32] consisted of a similar sample size (*n* = 25 vs. *n* = 24), with slightly higher baseline PASI scores (average 14.1 vs. 10.1), and comparable baseline PGA scores (average 2.7 vs. 3.1). The same statistical approach used for comparing nonlesional samples to lesional samples at baseline resulted in 4585 DEG. The two signatures shared approximately 62% of genes that were modulated in the same direction. Pathway enrichment analyses showed that 78% of the underlying pathways were shared (see Additional files 1 and 2). “Role of IL-17A in psoriasis” ranked high in both signatures, supporting its role in both populations studied.

### Gene expression signature of *Indigo naturalis* treatment

**Fig. 4:**
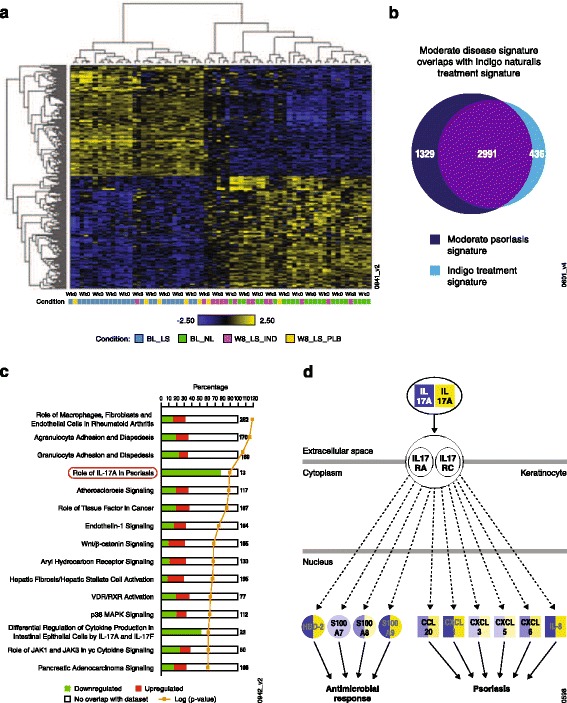
*Indigo naturalis* treatment signature. (**a**) Hierarchical clustering diagram of baseline and post-*Indigo naturalis* treatment (week 8) samples of the entire study cohort; genes are represented as rows and samples as columns. Upregulated genes are shown in *yellow* and downregulated in *blue*. Samples from four different groups are denoted as follows: baseline lesional (BL_LS; *blue*), baseline nonlesional (BL_NL; *green*), week-8 *Indigo naturalis*-treated lesional (W8_LS_IND; *purple*), and week-8 placebo-treated (W8_LS_PLB; *yellow*); (**b**) a Venn diagram comparing moderate psoriasis gene signature (*dark blue* circle on the left) and *Indigo naturalis* treatment signature (*light blue* circle on the right); (**c**) enrichment of Ingenuity pathways by *Indigo naturalis* treatment gene signature. The stacked bar chart displays the number of up-regulated (*red*) and down-regulated (*green*) in each Ingenuity Canonical Pathway. The pathways are ranked by the *p*-value of a Fisher exact test from top to bottom (orange line; *p*-value listed in Additional file 3); (**d**) the “Role of IL-17A in psoriasis” pathway overlaid with the gene expression pattern and each circle represents one gene. Differential expression between lesional and nonlesional sample at baseline is shown in left-half of each circle, and differential expression between week 8 and baseline samples is shown in right- half of circles. *Yellow* = up-regulated, *blue* = down-regulated, IL = interleukin, RA, receptor A, RC, receptor C

To investigate the pharmacological impact of *Indigo naturalis* treatment, gene expression patterns of post-treatment lesional skin were compared to pre-treatment lesional skin in the scope of the disease signature. By hierarchical clustering, 8/14 *Indigo naturalis*-treated week-8 samples were clustered together with baseline nonlesional samples, indicative of a return toward normal gene expression patterns, compared with only one placebo-treated sample (Fig. 4a). Five *Indigo naturalis* and two placebo-treated samples showed a mixed pattern between baseline lesional and nonlesional samples, indicating heterogeneity of the treatment effects.

**Fig. 5:**
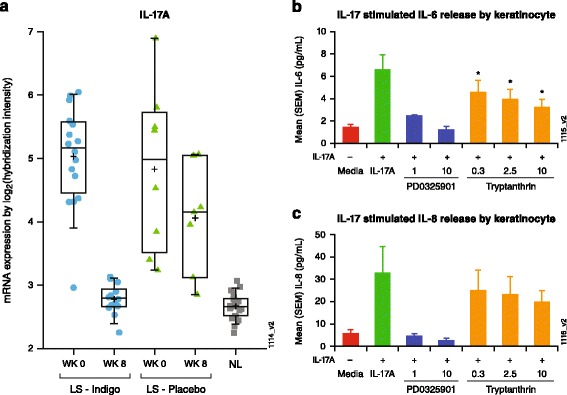
*Indigo naturalis*’ impact on the IL-17 pathway. (**a**) Down-regulation of interleukin (IL)-17A mRNA in the lesional skin (LS). *Indigo naturalis*-treated samples (*blue filled-circle*) are the left columns (week 0 [WK 0]: *n* = 16; week 8 [WK 8]: *n* = 14); placebo-treated samples (*green triangles*) are the middle two columns (WK 0: *n* = 8; WK 8: *n* = 7), and baseline nonlesional (NL) samples (gray square) are the right column (*n* = 23); (**b**) inhibition of IL-17-induced IL-6 secretion in cultured human keratinocytes. 100 mg/mL of IL-17 is present in all samples except media alone (first column). Compounds are tested in the following order: PD 0325901 1 μg (*n* = 2), 10 μg (*n* = 2); Tryptanthrin 0.3 μg (*n* = 7), 2.5 μg (*n* = 7) and 10 μg (*n* = 7). ^*^T-test *p*-value ≤0.05; (**c**) inhibition of IL-17-induced IL-8 secretion in cultured human keratinocytes. 100 mg/mL of IL-17 is present in all samples except media alone (first column). IL, interleukin; mRNA, messenger RNA; SEM, standard error of mean

A gene signature of *Indigo naturalis* treatment was generated by comparing week-8 lesional skin to the matched baseline samples from nine patients who achieved PASI 75 responses. The signature consisted of 5324 probe sets that corresponded to 3427 DEG (data not shown). When compared with the disease signature, 69% of genes were in common, but regulated in the opposite direction, indicating that a majority of the disease signature genes were impacted by the treatment (Fig. 4b). Enrichment analysis for this treatment signature also supports an impact on the majority of disease pathways (4 of 5 top pathways were identical to that of the disease signature, but in the opposite direction of gene regulation) (Fig. 4c; see Additional file 3). The pathway “Role of IL-17A in psoriasis” ranked even higher in this analysis than in the disease signature, and all pathway genes were reversely regulated (Fig. 4d). For example, IL-17A expression was decreased by 4.8-fold following *Indigo naturalis* treatment (*p* = 4.6 E-11) versus a non-significant 1.7-fold decrease with placebo treatment (Fig. 5a). Together, these data provide evidence that expression of IL-17A and its pathway genes was upregulated in psoriatic lesions of our study patients and down-regulated after *Indigo naturalis* treatment.

### Inhibition of IL-17-induced cytokine release by chemical compounds in *Indigo naturalis*

To further understand the mechanism of action, the three major chemical components of *Indigo naturalis*— isatin, indirubin, and tryptanthrin— were evaluated for IL-17 inhibition activity using an IL-17-induced cytokine release assay. Cultured human keratinocytes were stimulated with IL-17A (100 ng/mL), which resulted in a 4.5- and 5.5-fold increase in secretion of the pro-inflammatory cytokines IL-6 and IL-8, respectively. This elevation in production of these cytokines was inhibited by the MEK inhibitor PD 0325901 (Fig. 5b & c), but was not affected by the presence of isatin and indirubin (data not shown). On the contrary, tryptanthrin showed moderate, but significant inhibition, reducing IL-6 secretion by nearly half at the highest concentration tested (Fig. 5b). More moderate reduction of IL-17-stimulated IL-8 secretion was also observed (Fig. 5c).

## Discussion

*Indigo naturalis* is a TCM long recognized as a therapy for several inflammatory conditions [20, 36, 37]. Previous clinical studies have shown that monotherapy as a topical agent can be an effective treatment for mild-to-moderate psoriasis [17, 22]; however, interpretation of these studies is limited due to the lack of proper placebo controls. There also remains little understanding of the mechanism of action, although there is anecdotal evidence attributed to the anti-proliferation activity of indirubin [27]. Our study was designed to address these critical questions at the both the clinical and molecular level.

Previous clinical studies have included intra-patient placebo controls: placebo was applied to one side of the body, and *Indigo naturalis* to the other side of the same patient. Therefore, it was not administered in a truly blinded manner, and there was the possibility of exposure of active agent to the contra-lateral, placebo-exposed side via potential percutaneous absorption [17, 19]. To avoid bias, our study was designed as a double-blinded, placebo-control trial in which each patient was assigned to either active agent or placebo. Furthermore, the placebo was prepared using a blue color to match the active agent in appearance and to maintain the study blinding to prevent any possible bias.

We generated, for the first time, a comprehensive profile of molecular changes associated with moderate psoriasis and with the treatment effect of *Indigo naturalis* in skin. As in more severe disease, deregulation of IL-17 expression was observed in the more moderate population in this study. We presented evidence that topical *Indigo naturalis* significantly down-regulated the IL-17 pathway in affected skin, similar to other therapies that successfully target this pathway [9, 11, 38–40]. Moreover, we showed that one of the chemical components in *Indigo naturalis*, tryptanthrin, possesses moderate anti-IL-17 activity. To our knowledge, this is the first report that associates *Indigo naturalis*, in particular one of its chemical ingredients, tryptanthrin, with anti-IL-17 activity. Although an earlier study found that indirubin derivatives can inhibit STAT3 [41], which was shown to be a required transcription factor for Th17 differentiation [42], the link between indirubin-STAT3 and IL-17 has not been directly proven.

Although there has been a great deal of interest in understanding the pharmacologic mechanism of TCMs, limited progress has been made. One challenge is identifying the active components within TCM, as there may be numerous chemical components in a single TCM. Here, we have employed a different approach by first generating a treatment molecular signature and then identifying the specific pathways that are affected by TCM treatment, resulting in a hypothesis (IL-17 inhibition) that could be tested using purer chemical components.

We have made an effort to compare the disease signatures generated from our study population to a similar study [32] with a predominantly White population. The two signatures are mostly overlapping, indicating that the underlying driver of the disease may be the same in both psoriasis populations and suggesting that *Indigo naturalis* should be effective in broader populations of psoriasis patients.

There were a few limitations in this study, including small sample size and no systemic marker measurement. The latter limited the ability to address the reasons behind the short benefit duration of topical *Indigo naturalis* treatment. It is unknown whether this could be due to the lack of deeper skin penetration, leading to lower systemic exposure, or a relatively short half-life of the effective chemical ingredients. Future studies with a larger sample size and additional markers, such as serum tryptanthrin and IL-17 levels, would be more informative**.**

## Conclusion

In summary, this experimental medicine study in patients with moderate psoriasis identified IL-17 as a key pathway that can be modulated by treatment with *Indigo naturalis*. This study exemplified a novel approach to understanding the mechanism of action of a TCM, which may be applied to understanding the therapeutic effect of other forms of TCMs.

## Additional files

Additional file 1: Enrichment of ingenuity pathways by moderate psoriasis gene signature from our study population. (DOCX 22 kb)

Additional file 2: Enrichment of ingenuity pathways by gene signature from a predominately White psoriasis population [27]. (DOCX 18 kb)

Additional file 3: Enrichment of ingenuity pathways by gene signature of *Indigo naturalis* treatment of moderate psoriasis from our study population. (DOCX 23 kb)

## Abbreviations

BSA: Body surface area
DEG: differentially expressed genes
FTU: fingertip units
IL: Interleukin
MEK: mitogen-activated protein kinase kinase
OTPSS: Overall Target Plaque Severity Score
PASI 75: 75% improvement in PASI score from baseline
PASI: Psoriasis Area and Severity Index
PGA: Physician’s Global Assessment
RNA: ribonucleic acid
STAT-3: signal transducer and activator of transcription-3
TCM: Traditional Chinese Medicine
Th: T helper
TNF-α: tumor necrosis factor-alpha

## References

[1] Schön MP, Boehncke WH (2005). Psoriasis. N Engl J Med.

[2] Ding X, Wang T, Shen Y, Wang X, Zhou C, Tian S (2010). Prevalence of psoriasis in China: an epidemiological survey in six provinces. Chin J Derm Venereol.

[3] Liao H-T, Lin KC, Chang YT, Chen CH, Liang TH, Chen WS (2008). Human leukocyte antigen and clinical and demographic characteristics in psoriatic arthritis and psoriasis in Chinese patients. J Rheumatol.

[4] Zhang X, Wang H, Te-Shao H, Yang S, Chen S (2002). The genetic epidemiology of psoriasis vulgaris in Chinese Han. Int J Dermatol.

[5] Chiricozzi A, Guttman-Yassky E, Suárez-Fariñas M, Nograles KE, Tian S, Cardinale I (2011). Integrative responses to IL-17 and TNF-α in human keratinocytes account for key inflammatory pathogenic circuits in psoriasis. J Invest Dermatol.

[6] Leonardi CL, Kimball AB, Papp KA, Yeilding N, Guzzo C, Wang Y (2008). PHOENIX 1 study investigators. Efficacy and safety of ustekinumab, a human interleukin-12/23 monoclonal antibody, in patients with psoriasis: 76-week results from a randomised, double-blind, placebo-controlled trial (PHOENIX 1). Lancet.

[7] Papp KA, Langley RG, Lebwohl M, Krueger GG, Szapary P, Yeilding N (2008). PHOENIX 2 study investigators. Efficacy and safety of ustekinumab, a human interleukin-12/23 monoclonal antibody, in patients with psoriasis: 52-week results from a randomised, double-blind, placebo-controlled trial (PHOENIX 2). Lancet.

[8] Sofen H, Smith S, Matheson RT, Leonardi CL, Calderon C, Brodmerkel C (2014). Guselkumab (an IL-23–specific mAb) demonstrates clinical and molecular response in patients with moderate-to-severe psoriasis. J Allergy Clin Immunol.

[9] Zaba LC, Suárez-Fariñas M, Fuentes-Duculan J, Nograles KE, Guttman-Yassky E, Cardinale I, et al. Effective treatment of psoriasis with etanercept is linked to suppression of IL-17 signaling, not immediate response TNF genes.J Allergy Clin Immunol 2009;124:1022–10.e1–395. 10.1016/j.jaci.2009.08.046 PMC2852188 19895991

[10] Krueger JG, Fretzin S, Suárez-Fariñas M, Haslett PA, Phipps KM, Cameron GS (2012). IL-17A is essential for cell activation and inflammatory gene circuits in psoriasis. J Allergy Clin Immunol.

[11] Balato A, Schiattarella M, Di Caprio R, Lembo S, Mattii M, Balato N (2014). Effects of adalimumab therapy in adult subjects with moderate-to-severe psoriasis on Th17 pathway. J Eur Acad Dermatol Venereol.

[12] Russell CB, Rand H, Bigler J, Kerkof K, Timour M, Bautista E (2014). Gene expression profiles normalized in psoriatic skin by treatment with brodalumab, a human anti–IL-17 receptor monoclonal antibody. J Immunol.

[13] Mason AR, Mason JM, Cork MJ, Hancock H, Dooley G (2013). Topical treatments for chronic plaque psoriasis of the scalp: a systematic review. Br J Dermatol.

[14] Koo J, Arain S (1998). Traditional Chinese medicine for the treatment of dermatologic disorders. Arch Dermatol.

[15] Tse TW (2003). Use of common Chinese herbs in the treatment of psoriasis. Clin Exp Dermatol.

[16] Tse WP, Che CT, Liu K, Lin ZX (2006). Evaluation of the anti-proliferative properties of selected psoriasis-treating Chinese medicines on cultured HaCaT cells. J Ethnopharmacol.

[17] Lin YK, Wong WR, Chang YC, Chang CJ, Tsay PK, Chang SC (2007). The efficacy and safety of topically applied indigo naturalis ointment in patients with plaque-type psoriasis. Dermatology.

[18] Lin YK, Wong WR, Su Pang JH (2007). Successful treatment of recalcitrant psoriasis with indigo naturalis ointment. Clin Exp Dermatol.

[19] Lin YK, Chang CJ, Chang YC, Wong WR, Chang SC, Pang JH (2008). Clinical assessment of patients with recalcitrant psoriasis in a randomized, observer-blind, vehicle-controlled trial using indigo naturalis. Arch Dermatol.

[20] Lin YK, Leu YL, Yang SH, Chen HW, Wang CT, Pang JH (2009). Anti-psoriatic effects of indigo naturalis on the proliferation and differentiation of keratinocytes with indirubin as the active component. J Dermatol Sci.

[21] Lin YK, Leu YL, Huang TH, Wu YH, Chung PJ, Su Pang JH, et al. Anti-inflammatory effects of the extract of indigo naturalis in human neutrophils. J Ethnopharmacol. 2009;25:51–8. 10.1016/j.jep.2009.06.014 19559779

[22] Lin YK, Leu YL, Huang TH, Wu YH, Chung PJ, Su Pang JH, et al. Comparison of refined and crude indigo naturalis ointment in treating psoriasis: randomized, observer-blind, controlled, intrapatient trial. Arch Dermatol. 2012;148:397–400. 10.1001/archdermatol.2011.1091 22431789

[23] Ports WC, Khan S, Lan S, Lamba M, Bolduc C, Bissonnette R (2015). Randomized pilot clinical trial of tofacitinib solution for plaque psoriasis: challenges of the intra-subject study design. J Drugs Dermatol.

[24] Hoessel R, Leclerc S, Endicott JA, Nobel ME, Lawrie A, Tunnah P, Leost M (1999). Indirubin, the active constituent of a Chinese antileukaemia medicine, inhibits cyclin-dependent kinases. Nature Cell Biol.

[25] Leclerc S, Garnier M, Hoessel R, Marko D, Bibb JA, Snyder GL (2001). Indirubins inhibit glycogen synthase kinase-3 β and CDK5/p25, two protein kinases involved in abnormal tau phosphorylation in Alzheimer's disease. A property common to most cyclin-dependent kinase inhibitors?. J Biol Chem.

[26] Schwaiberger AV, Heiss EH, Cabaravdic M, Oberan T, Zaujec J, Schachner D, et al. Indirubin-3′-monoxime blocks vascular smooth muscle cell proliferation by inhibition of signal transducer and activator of transcription 3 signaling and reduces neointima formation in vivo. Arterioscler Thromb Vasc Biol. 2010;30:2475–81. 10.1161/ATVBAHA.110.212654 20847306

[27] Hsieh WL, Lin YK, Tsai CN, Wang TM, Chen TY, Pang JH (2012). Indirubin, an acting component of indigo naturalis, inhibits EGFR activation and EGF-induced CDC25B gene expression in epidermal keratinocytes. J Dermatol Sci.

[28] Takei Y, Kunikata T, Aga M, Inoue S, Ushio S, Iwaki K (2003). Tryptanthrin inhibits interferon-γ production by Peyer's patch lymphocytes derived from mice that had been orally administered staphylococcal enterotoxin. Biol Pharm Bull.

[29] Ishihara T, Kohno K, Ushio S, Iwaki K, Ikeda M, Kurimoto M (2000). Tryptanthrin inhibits nitric oxide and prostaglandin E_2_ synthesis by murine macrophages. Eur J Pharmacol.

[30] Reuter J, Wölfle U, Weckesser S, Schempp C (2010). Which plant for which skin disease? Part 1: atopic dermatitis, psoriasis, acne, condyloma and herpes simplex. J Dtsch Dermatol Ges.

[31] Bernstein S, Donsky H, Gulliver W, Hamilton D, Nobel S, Norman R (2006). Treatment of mild to moderate psoriasis with Relieva, a Mahonia Aquifolium extract—a double-blind, placebo-controlled study. Am J Ther.

[32] Papp KA, Reid C, Foley P, Sinclair R, Salinger DH, Williams G, et al. Anti-IL-17 receptor antibody AMG 827 leads to rapid clinical response in subjects with moderate to severe psoriasis: results from a phase I, randomized, placebo-controlled trial. J Invest Dermatol. 2012;132:2466–9. 10.1038/jid.2012.163 22622425

[33] Barrett SD, Bridges AJ, Dudley DT, Saltiel AR, Fergus JH, Flamme CM (2008). The discovery of the benzhydroxamate MEK inhibitors CI-1040 and PD 0325901. Bioorg Med Chem Lett.

[34] Laan M, Lötvall J, Chung KF, Lindén A (2001). IL-17-induced cytokine release in human bronchial epithelial cells in vitro: role of mitogen-activated protein (MAP) kinases. Br J Pharmacol.

[35] Langley RG, Ellis CN (2004). Evaluating psoriasis with psoriasis area and severity index, psoriasis global assessment and lattice system Physician's global assessment. J Am Acad Dermatol.

[36] Kim MH, Choi YY, Yang G, Cho IH, Nam D, Yang WM (2013). Indirubin, a purple 3, 2-bisindole, inhibited allergic contact dermatitis via regulating T helper (Th)-mediated immune system in DNCB-induced model. J Ethnopharmacol.

[37] Xiao HT, Peng J, Hu DD, Lin CY, Du B, Tsang SW (2015). Qing-dai powder promotes recovery of colitis by inhibiting inflammatory responses of colonic macrophages in dextran sulfate sodium-treated mice. Chin Med.

[38] Papp KA, Langley RG, Sigurgeirsson B, Abe M, Baker DR, Konno P (2013). Efficacy and safety of secukinumab in the treatment of moderate-to-severe plaque psoriasis: a randomized, double-blind, placebo-controlled phase II dose-ranging study. Br J Dermatol.

[39] Papp K, Leonardi C, Menter A, Ortonne JP, Krueger JG, Kricorian G (2012). Brodalumab, an anti–interleukin-17–receptor antibody for psoriasis. N Engl J Med.

[40] Gordon KB, Blauvelt A, Papp KA, Langley RG, Luger T, Ohtsuki M (2016). UNCOVER-1 study group; UNCOVER-2 study group; UNCOVER-3 study group. Phase 3 trials of ixekizumab in moderate-to-severe plaque psoriasis. N Engl J Med.

[41] Nam S, Buettner R, Turkson J, Kim D, Cheng JQ, Muehlbeyer S (2005). Indirubin derivatives inhibit Stat3 signaling and induce apoptosis in human cancer cells. Proc Natl Acad Sci U S A.

[42] Harris TJ, Grosso JF, Yen HR, Xin H, Kortylewski M, Albesiano E (2007). An in vivo requirement for STAT3 signaling in TH17 development and TH17-dependent autoimmunity. J Immunol.

