# Supplementary material for: Clinical efficacy and IL-17 targeting mechanism of Indigo naturalis as a topical agent in moderate psoriasis
Source: BMC Complement Altern Med. 2017 Sep 2;17:439. doi: 10.1186/s12906-017-1947-1 (PMC5581407; doi:10.1186/s12906-017-1947-1)
Supplement: Supplementary file 1 — Enrichment of ingenuity pathways by moderate psoriasis gene signature from our study population. (DOCX 22 kb) [file 12906_2017_1947_MOESM1_ESM.docx]

**Additional file 1. Enrichment of ingenuity pathways by moderate psoriasis gene signature from our study population**

| **Ingenuity Canonical Pathways** | **-log(p-value)** | **Ratio** |
| --- | --- | --- |
| Granulocyte Adhesion and Diapedesis | 9.52 | 0.43 |
| Agranulocyte Adhesion and Diapedesis | 9.47 | 0.42 |
| Atherosclerosis Signaling | 7.29 | 0.43 |
| Role of Macrophages, Fibroblasts and Endothelial Cells in Rheumatoid Arthritis | 6.77 | 0.35 |
| Graft-versus-Host Disease Signaling | 6.51 | 0.57 |
| Altered T Cell and B Cell Signaling in Rheumatoid Arthritis | 6.05 | 0.46 |
| Protein Ubiquitination Pathway | 5.89 | 0.34 |
| LPS/IL-1 Mediated Inhibition of RXR Function | 5.80 | 0.36 |
| Role of IL-17A in Psoriasis | 5.67 | 0.85 |
| Type I Diabetes Mellitus Signaling | 5.65 | 0.42 |
| TREM1 Signaling | 5.50 | 0.46 |
| Dendritic Cell Maturation | 5.46 | 0.37 |
| Hepatic Fibrosis / Hepatic Stellate Cell Activation | 4.96 | 0.35 |
| Interferon Signaling | 4.94 | 0.56 |
| T Helper Cell Differentiation | 4.82 | 0.45 |
| NF-ΚB Signaling | 4.80 | 0.36 |
| Role of NFAT in Regulation of the Immune Response | 4.74 | 0.36 |
| Production of Nitric Oxide and Reactive Oxygen Species in Macrophages | 4.56 | 0.35 |
| Pathogenesis of Multiple Sclerosis | 4.50 | 0.89 |
| Role of Tissue Factor in Cancer | 4.33 | 0.38 |
| Communication between Innate and Adaptive Immune Cells | 4.08 | 0.40 |
| Role of Hypercytokinemia/hyperchemokinemia in the Pathogenesis of Influenza | 4.04 | 0.49 |
| Molecular Mechanisms of Cancer | 4.02 | 0.30 |
| p38 MAPK Signaling | 4.01 | 0.37 |
| Role of JAK1 and JAK3 in γc Cytokine Signaling | 3.96 | 0.43 |
| PPAR Signaling | 3.93 | 0.39 |
| Hereditary Breast Cancer Signaling | 3.81 | 0.37 |
| IL-10 Signaling | 3.76 | 0.41 |
| iNOS Signaling | 3.68 | 0.47 |
| Antigen Presentation Pathway | 3.67 | 0.49 |
| IL-17 Signaling | 3.67 | 0.40 |
| Aryl Hydrocarbon Receptor Signaling | 3.67 | 0.35 |
| ATM Signaling | 3.65 | 0.42 |
| Xenobiotic Metabolism Signaling | 3.64 | 0.31 |
| Differential Regulation of Cytokine Production in Intestinal Epithelial Cells by IL-17A and IL-17F | 3.61 | 0.57 |
| Fcγ Receptor-mediated Phagocytosis in Macrophages and Monocytes | 3.61 | 0.38 |
| Axonal Guidance Signaling | 3.49 | 0.28 |
| Role of BRCA1 in DNA Damage Response | 3.42 | 0.41 |
| Nur77 Signaling in T Lymphocytes | 3.41 | 0.43 |
| Ephrin Receptor Signaling | 3.38 | 0.33 |
| Tumoricidal Function of Hepatic Natural Killer Cells | 3.37 | 0.54 |
| ErbB Signaling | 3.35 | 0.38 |
| Glycogen Degradation II | 3.31 | 0.78 |
| Wnt/β-catenin Signaling | 3.27 | 0.33 |
| Toll-like Receptor Signaling | 3.27 | 0.39 |
| DNA damage-induced 14-3-3σ Signaling | 3.25 | 0.58 |
| Endothelin-1 Signaling | 3.20 | 0.32 |
| IL-4 Signaling | 3.16 | 0.38 |
| Role of Osteoblasts, Osteoclasts and Chondrocytes in Rheumatoid Arthritis | 3.14 | 0.31 |
| Mitotic Roles of Polo-Like Kinase | 3.13 | 0.40 |
| B Cell Development | 3.12 | 0.50 |
| IL-6 Signaling | 3.12 | 0.35 |
| Death Receptor Signaling | 3.10 | 0.36 |
| VDR/RXR Activation | 3.10 | 0.38 |
| Glucocorticoid Receptor Signaling | 3.09 | 0.30 |
| CD28 Signaling in T Helper Cells | 3.07 | 0.35 |
| Pancreatic Adenocarcinoma Signaling | 3.04 | 0.35 |
| IL-17A Signaling in Airway Cells | 3.01 | 0.39 |
| iCOS-iCOSL Signaling in T Helper Cells | 2.99 | 0.35 |
| Inhibition of Angiogenesis by TSP1 | 2.94 | 0.47 |
| Adipogenesis pathway | 2.91 | 0.33 |
| Role of CHK Proteins in Cell Cycle Checkpoint Control | 2.88 | 0.40 |
| Role of Cytokines in Mediating Communication between Immune Cells | 2.83 | 0.40 |
| PPARα/RXRα Activation | 2.83 | 0.32 |
| Mouse Embryonic Stem Cell Pluripotency | 2.83 | 0.35 |
| IL-12 Signaling and Production in Macrophages | 2.82 | 0.33 |
| RAR Activation | 2.81 | 0.31 |
| NRF2-mediated Oxidative Stress Response | 2.80 | 0.31 |
| Salvage Pathways of Pyrimidine Deoxyribonucleotides | 2.75 | 0.75 |
| MSP-RON Signaling Pathway | 2.75 | 0.41 |
| Estrogen-mediated S-phase Entry | 2.75 | 0.50 |
| Aldosterone Signaling in Epithelial Cells | 2.74 | 0.32 |
| LXR/RXR Activation | 2.73 | 0.33 |
| p70S6K Signaling | 2.67 | 0.33 |
| Tec Kinase Signaling | 2.67 | 0.31 |
| Glioblastoma Multiforme Signaling | 2.63 | 0.32 |
| Role of Pattern Recognition Receptors in Recognition of Bacteria and Viruses | 2.60 | 0.33 |
| Role of IL-17A in Arthritis | 2.59 | 0.39 |
| Glycogen Degradation III | 2.53 | 0.64 |
| Role of NANOG in Mammalian Embryonic Stem Cell Pluripotency | 2.50 | 0.33 |
| p53 Signaling | 2.49 | 0.34 |
| CTLA4 Signaling in Cytotoxic T Lymphocytes | 2.49 | 0.35 |
| IL-17A Signaling in Fibroblasts | 2.46 | 0.43 |
| IL-8 Signaling | 2.45 | 0.30 |
| IL-15 Signaling | 2.43 | 0.36 |
| Autoimmune Thyroid Disease Signaling | 2.41 | 0.41 |
| Cell Cycle: G2/M DNA Damage Checkpoint Regulation | 2.38 | 0.39 |
| Hypoxia Signaling in the Cardiovascular System | 2.37 | 0.37 |
| Docosahexaenoic Acid (DHA) Signaling | 2.36 | 0.41 |
| Prostanoid Biosynthesis | 2.36 | 0.67 |
| TR/RXR Activation | 2.35 | 0.34 |
| ILK Signaling | 2.34 | 0.30 |
| Primary Immunodeficiency Signaling | 2.33 | 0.39 |
| Sphingosine-1-phosphate Signaling | 2.30 | 0.32 |
| FLT3 Signaling in Hematopoietic Progenitor Cells | 2.30 | 0.35 |
| eNOS Signaling | 2.29 | 0.31 |
| Glioma Invasiveness Signaling | 2.27 | 0.37 |
| Cholecystokinin/Gastrin-mediated Signaling | 2.26 | 0.33 |
| Retinoate Biosynthesis I | 2.25 | 0.43 |
| Eicosanoid Signaling | 2.22 | 0.36 |
| PKCθ¸ Signaling in T Lymphocytes | 2.22 | 0.32 |
| Tryptophan Degradation to 2-amino-3-carboxymuconate Semialdehyde | 2.20 | 0.71 |
| Triacylglycerol Degradation | 2.19 | 0.46 |
| HER-2 Signaling in Breast Cancer | 2.18 | 0.34 |
| Differential Regulation of Cytokine Production in Macrophages and T Helper Cells by IL-17A and IL-17F | 2.17 | 0.50 |
| Calcium-induced T Lymphocyte Apoptosis | 2.17 | 0.36 |
| IL-9 Signaling | 2.15 | 0.41 |
| HMGB1 Signaling | 2.14 | 0.31 |
| Role of IL-17F in Allergic Inflammatory Airway Diseases | 2.11 | 0.39 |
| Allograft Rejection Signaling | 2.11 | 0.38 |
| Cytotoxic T Lymphocyte-mediated Apoptosis of Target Cells | 2.10 | 0.42 |
| Role of MAPK Signaling in the Pathogenesis of Influenza | 2.09 | 0.35 |
| T Cell Receptor Signaling | 2.08 | 0.32 |
| Inhibition of Matrix Metalloproteases | 2.07 | 0.40 |
| Crosstalk between Dendritic Cells and Natural Killer Cells | 2.04 | 0.33 |
| IL-17A Signaling in Gastric Cells | 2.03 | 0.44 |
| IGF-1 Signaling | 2.01 | 0.32 |
| NAD biosynthesis II (from tryptophan) | 1.99 | 0.54 |
| GADD45 Signaling | 1.98 | 0.47 |
| Acute Phase Response Signaling | 1.98 | 0.29 |
| Granzyme B Signaling | 1.97 | 0.50 |
| Unfolded protein response | 1.96 | 0.36 |
| IL-2 Signaling | 1.96 | 0.36 |
| Glioma Signaling | 1.95 | 0.32 |
| PTEN Signaling | 1.90 | 0.31 |
| Ovarian Cancer Signaling | 1.89 | 0.30 |
| Growth Hormone Signaling | 1.84 | 0.33 |
| Prolactin Signaling | 1.82 | 0.33 |
| STAT3 Pathway | 1.82 | 0.33 |
| Retinol Biosynthesis | 1.80 | 0.40 |
| DNA Double-Strand Break Repair by Homologous Recombination | 1.78 | 0.50 |
| Factors Promoting Cardiogenesis in Vertebrates | 1.77 | 0.32 |
| VEGF Signaling | 1.77 | 0.32 |
| Salvage Pathways of Pyrimidine Ribonucleotides | 1.76 | 0.31 |
| Sertoli Cell-Sertoli Cell Junction Signaling | 1.74 | 0.28 |
| Oncostatin M Signaling | 1.73 | 0.38 |
| ERK5 Signaling | 1.73 | 0.33 |
| Colorectal Cancer Metastasis Signaling | 1.72 | 0.27 |
| Neurotrophin/TRK Signaling | 1.71 | 0.33 |
| EGF Signaling | 1.69 | 0.34 |
| Superoxide Radicals Degradation | 1.67 | 0.67 |
| UVA-Induced MAPK Signaling | 1.65 | 0.31 |
| Clathrin-mediated Endocytosis Signaling | 1.61 | 0.28 |
| VEGF Family Ligand-Receptor Interactions | 1.61 | 0.32 |
| Prostate Cancer Signaling | 1.60 | 0.31 |
| Leukocyte Extravasation Signaling | 1.58 | 0.28 |
| Role of JAK family kinases in IL-6-type Cytokine Signaling | 1.57 | 0.40 |
| Glutathione-mediated Detoxification | 1.57 | 0.40 |
| Pyrimidine Ribonucleotides Interconversion | 1.57 | 0.40 |
| Noradrenaline and Adrenaline Degradation | 1.56 | 0.38 |
| Leptin Signaling in Obesity | 1.55 | 0.32 |
| Phospholipases | 1.55 | 0.33 |
| Cyclins and Cell Cycle Regulation | 1.54 | 0.31 |
| ERK/MAPK Signaling | 1.53 | 0.27 |
| Virus Entry via Endocytic Pathways | 1.52 | 0.30 |
| FXR/RXR Activation | 1.52 | 0.29 |
| Estrogen-Dependent Breast Cancer Signaling | 1.52 | 0.32 |
| GM-CSF Signaling | 1.52 | 0.32 |
| PI3K/AKT Signaling | 1.51 | 0.29 |
| Thrombin Signaling | 1.49 | 0.27 |
| Creatine-phosphate Biosynthesis | 1.48 | 0.75 |
| NAD Biosynthesis III | 1.48 | 0.75 |
| Role of PKR in Interferon Induction and Antiviral Response | 1.48 | 0.35 |
| Thrombopoietin Signaling | 1.47 | 0.33 |
| Circadian Rhythm Signaling | 1.46 | 0.36 |
| Pyridoxal 5'-phosphate Salvage Pathway | 1.44 | 0.32 |
| Cell Cycle: G1/S Checkpoint Regulation | 1.44 | 0.32 |
| Gap Junction Signaling | 1.44 | 0.28 |
| RAN Signaling | 1.43 | 0.44 |
| Putrescine Degradation III | 1.43 | 0.44 |
| Parkinson's Signaling | 1.43 | 0.44 |
| IL-3 Signaling | 1.43 | 0.31 |
| Small Cell Lung Cancer Signaling | 1.43 | 0.31 |
| Reelin Signaling in Neurons | 1.42 | 0.30 |
| TGF-β Signaling | 1.41 | 0.30 |
| Ethanol Degradation II | 1.40 | 0.37 |
| Choline Biosynthesis III | 1.39 | 0.46 |
| AMPK Signaling | 1.38 | 0.28 |
| Chemokine Signaling | 1.37 | 0.31 |
| Acute Myeloid Leukemia Signaling | 1.36 | 0.30 |
| Natural Killer Cell Signaling | 1.36 | 0.29 |
| HIF1α Signaling | 1.35 | 0.29 |
| β-alanine Degradation I | 1.34 | 1.00 |
| Guanine and Guanosine Salvage I | 1.34 | 1.00 |
| Choline Degradation I | 1.34 | 1.00 |
| Pyrimidine Ribonucleotides De Novo Biosynthesis | 1.34 | 0.37 |
| UVB-Induced MAPK Signaling | 1.34 | 0.32 |
| Cdc42 Signaling | 1.33 | 0.28 |
| Hepatic Cholestasis | 1.32 | 0.27 |
| Ephrin B Signaling | 1.30 | 0.30 |
| Regulation of eIF4 and p70S6K Signaling | 1.30 | 0.28 |
| PDGF Signaling | 1.30 | 0.30 |
| IL-22 Signaling | 1.30 | 0.38 |
