# Supplementary material for: Clinical efficacy and IL-17 targeting mechanism of Indigo naturalis as a topical agent in moderate psoriasis
Source: BMC Complement Altern Med. 2017 Sep 2;17:439. doi: 10.1186/s12906-017-1947-1 (PMC5581407; doi:10.1186/s12906-017-1947-1)
Supplement: Supplementary file 2 — Enrichment of ingenuity pathways by gene signature from a predominately White psoriasis population [27]. (DOCX 18 kb) [file 12906_2017_1947_MOESM2_ESM.docx]

**Additional file 2. Enrichment of ingenuity pathways by gene signature from a predominately White psoriasis population [27]**

| **Ingenuity Canonical Pathways** | **-log(p-value)** | **Ratio** |
| --- | --- | --- |
| Agranulocyte Adhesion and Diapedesis | 6.9 | 0.43 |
| Granulocyte Adhesion and Diapedesis | 5.91 | 0.42 |
| Atherosclerosis Signaling | 4.05 | 0.41 |
| Role of IL-17A in Psoriasis | 3.95 | 0.77 |
| Fcγ Receptor-mediated Phagocytosis in Macrophages and Monocytes | 3.85 | 0.42 |
| LPS/IL-1 Mediated Inhibition of RXR Function | 3.61 | 0.36 |
| Estrogen-mediated S-phase Entry | 3.35 | 0.58 |
| Cardiac β-adrenergic Signaling | 3.3 | 0.38 |
| Cell Cycle Control of Chromosomal Replication | 3.24 | 0.56 |
| Ethanol Degradation II | 3.16 | 0.53 |
| Sperm Motility | 3.04 | 0.39 |
| Differential Regulation of Cytokine Production in Intestinal Epithelial Cells by IL-17A and IL-17F | 2.97 | 0.57 |
| Aryl Hydrocarbon Receptor Signaling | 2.94 | 0.37 |
| Pathogenesis of Multiple Sclerosis | 2.91 | 0.78 |
| Axonal Guidance Signaling | 2.88 | 0.31 |
| Nitric Oxide Signaling in the Cardiovascular System | 2.85 | 0.39 |
| Ephrin Receptor Signaling | 2.77 | 0.35 |
| Inhibition of Angiogenesis by TSP1 | 2.77 | 0.50 |
| Noradrenaline and Adrenaline Degradation | 2.77 | 0.50 |
| VDR/RXR Activation | 2.74 | 0.40 |
| Ethanol Degradation IV | 2.69 | 0.58 |
| Glutathione-mediated Detoxification | 2.6 | 0.55 |
| Role of Macrophages, Fibroblasts and Endothelial Cells in Rheumatoid Arthritis | 2.6 | 0.32 |
| Fatty Acid α-oxidation | 2.42 | 0.60 |
| Oxidative Ethanol Degradation III | 2.42 | 0.60 |
| Nicotine Degradation II | 2.4 | 0.43 |
| Endothelin-1 Signaling | 2.34 | 0.34 |
| Estrogen Biosynthesis | 2.27 | 0.46 |
| Role of Hypercytokinemia/hyperchemokinemia in the Pathogenesis of Influenza | 2.26 | 0.44 |
| Xenobiotic Metabolism Signaling | 2.22 | 0.32 |
| Hepatic Fibrosis / Hepatic Stellate Cell Activation | 2.21 | 0.33 |
| Bupropion Degradation | 2.19 | 0.50 |
| Pancreatic Adenocarcinoma Signaling | 2.18 | 0.36 |
| RAN Signaling | 2.17 | 0.56 |
| Granzyme B Signaling | 2.17 | 0.56 |
| Putrescine Degradation III | 2.17 | 0.56 |
| Retinoate Biosynthesis I | 2.14 | 0.47 |
| Thyroid Cancer Signaling | 2.13 | 0.44 |
| Role of Tissue Factor in Cancer | 2.11 | 0.36 |
| Role of CHK Proteins in Cell Cycle Checkpoint Control | 2.06 | 0.40 |
| Phospholipases | 2.05 | 0.40 |
| Clathrin-mediated Endocytosis Signaling | 2.04 | 0.33 |
| Cell Cycle: G2/M DNA Damage Checkpoint Regulation | 2.03 | 0.41 |
| Pyrimidine Ribonucleotides Interconversion | 2.02 | 0.48 |
| Acetone Degradation I (to Methylglyoxal) | 2.02 | 0.48 |
| Interferon Signaling | 1.99 | 0.44 |
| ATM Signaling | 1.98 | 0.39 |
| Tryptophan Degradation X (Mammalian, via Tryptamine) | 1.95 | 0.53 |
| Wnt/β-catenin Signaling | 1.93 | 0.33 |
| Serotonin Degradation | 1.92 | 0.40 |
| Tryptophan Degradation to 2-amino-3-carboxymuconate Semialdehyde | 1.92 | 0.71 |
| CCR5 Signaling in Macrophages | 1.91 | 0.38 |
| Mitotic Roles of Polo-Like Kinase | 1.91 | 0.38 |
| eNOS Signaling | 1.9 | 0.34 |
| Epithelial Adherens Junction Signaling | 1.9 | 0.33 |
| Gαi Signaling | 1.88 | 0.34 |
| Histamine Degradation | 1.88 | 0.58 |
| Inhibition of Matrix Metalloproteases | 1.86 | 0.42 |
| Creatine-phosphate Biosynthesis | 1.83 | 0.80 |
| Remodeling of Epithelial Adherens Junctions | 1.82 | 0.38 |
| Differential Regulation of Cytokine Production in Macrophages and T Helper Cells by IL-17A and IL-17F | 1.75 | 0.50 |
| Netrin Signaling | 1.75 | 0.41 |
| Pyrimidine Deoxyribonucleotides De Novo Biosynthesis I | 1.73 | 0.48 |
| cAMP-mediated signaling | 1.72 | 0.31 |
| Pyrimidine Ribonucleotides De Novo Biosynthesis | 1.71 | 0.44 |
| IL-12 Signaling and Production in Macrophages | 1.67 | 0.33 |
| Nicotine Degradation III | 1.65 | 0.40 |
| Gap Junction Signaling | 1.65 | 0.32 |
| NAD biosynthesis II (from tryptophan) | 1.65 | 0.54 |
| Choline Biosynthesis III | 1.65 | 0.54 |
| STAT3 Pathway | 1.62 | 0.36 |
| Relaxin Signaling | 1.61 | 0.33 |
| Tight Junction Signaling | 1.61 | 0.32 |
| Superpathway of Melatonin Degradation | 1.6 | 0.38 |
| Superoxide Radicals Degradation | 1.59 | 0.63 |
| Salvage Pathways of Pyrimidine Deoxyribonucleotides | 1.59 | 0.63 |
| DNA damage-induced 14-3-3σSignaling | 1.58 | 0.47 |
| IL-17A Signaling in Gastric Cells | 1.57 | 0.44 |
| Role of MAPK Signaling in the Pathogenesis of Influenza | 1.54 | 0.36 |
| G Beta Gamma Signaling | 1.53 | 0.34 |
| Molecular Mechanisms of Cancer | 1.5 | 0.29 |
| α-Adrenergic Signaling | 1.5 | 0.34 |
| Antioxidant Action of Vitamin C | 1.49 | 0.34 |
| Macropinocytosis Signaling | 1.49 | 0.35 |
| tRNA Splicing | 1.49 | 0.40 |
| Bladder Cancer Signaling | 1.43 | 0.34 |
| Dopamine-DARPP32 Feedback in cAMP Signaling | 1.43 | 0.31 |
| Dopamine Degradation | 1.42 | 0.45 |
| Leptin Signaling in Obesity | 1.37 | 0.34 |
| p38 MAPK Signaling | 1.35 | 0.32 |
| Oleate Biosynthesis II (Animals) | 1.33 | 0.56 |
| Glycogen Degradation II | 1.33 | 0.56 |
| Role of JAK1 and JAK3 in γc Cytokine Signaling | 1.32 | 0.35 |
| Protein Kinase A Signaling | 1.31 | 0.29 |
| Arsenate Detoxification I (Glutaredoxin) | 1.31 | 0.75 |
| Melatonin Degradation II | 1.31 | 0.75 |
| Arginine Degradation I (Arginase Pathway) | 1.31 | 0.75 |
| IL-22 Signaling | 1.3 | 0.42 |
| Tumoricidal Function of Hepatic Natural Killer Cells | 1.3 | 0.42 |
