# Supplementary material for: Clinical efficacy and IL-17 targeting mechanism of Indigo naturalis as a topical agent in moderate psoriasis
Source: BMC Complement Altern Med. 2017 Sep 2;17:439. doi: 10.1186/s12906-017-1947-1 (PMC5581407; doi:10.1186/s12906-017-1947-1)
Supplement: Supplementary file 3 — Enrichment of ingenuity pathways by gene signature of Indigo naturalis treatment of moderate psoriasis from our study population. (DOCX 23 kb) [file 12906_2017_1947_MOESM3_ESM.docx]

**Additional file 3. Enrichment of ingenuity pathways by gene signature of *Indigo naturalis* treatment of moderate psoriasis from our study population**

| **Ingenuity Canonical Pathways** | **-log(p-value)** | **Ratio** |
| --- | --- | --- |
| Role of Macrophages, Fibroblasts and Endothelial Cells in Rheumatoid Arthritis | 6.69 | 0.32 |
| Agranulocyte Adhesion and Diapedesis | 6.51 | 0.35 |
| Granulocyte Adhesion and Diapedesis | 5.98 | 0.35 |
| Role of IL-17A in Psoriasis | 5.03 | 0.77 |
| Atherosclerosis Signaling | 4.96 | 0.36 |
| Role of Tissue Factor in Cancer | 4.83 | 0.36 |
| Endothelin-1 Signaling | 4.24 | 0.32 |
| Wnt/β-catenin Signaling | 3.86 | 0.31 |
| Aryl Hydrocarbon Receptor Signaling | 3.83 | 0.32 |
| Hepatic Fibrosis / Hepatic Stellate Cell Activation | 3.79 | 0.30 |
| VDR/RXR Activation | 3.62 | 0.36 |
| p38 MAPK Signaling | 3.59 | 0.33 |
| Differential Regulation of Cytokine Production in Intestinal Epithelial Cells by IL-17A and IL-17F | 3.46 | 0.52 |
| Role of JAK1 and JAK3 in γc Cytokine Signaling | 3.46 | 0.38 |
| Pancreatic Adenocarcinoma Signaling | 3.43 | 0.33 |
| Axonal Guidance Signaling | 3.24 | 0.25 |
| ErbB Signaling | 3.20 | 0.34 |
| Ephrin Receptor Signaling | 3.11 | 0.29 |
| NF-ΚB Signaling | 3.08 | 0.29 |
| IL-17A Signaling in Airway Cells | 3.00 | 0.36 |
| IL-17 Signaling | 2.96 | 0.35 |
| Mouse Embryonic Stem Cell Pluripotency | 2.92 | 0.33 |
| PPAR Signaling | 2.76 | 0.32 |
| ATM Signaling | 2.73 | 0.36 |
| Mitotic Roles of Polo-Like Kinase | 2.71 | 0.35 |
| Role of Hypercytokinemia/hyperchemokinemia in the Pathogenesis of Influenza | 2.67 | 0.39 |
| Interferon Signaling | 2.65 | 0.41 |
| Role of NANOG in Mammalian Embryonic Stem Cell Pluripotency | 2.62 | 0.31 |
| Fcγ Receptor-mediated Phagocytosis in Macrophages and Monocytes | 2.59 | 0.32 |
| HER-2 Signaling in Breast Cancer | 2.59 | 0.33 |
| Human Embryonic Stem Cell Pluripotency | 2.55 | 0.29 |
| Factors Promoting Cardiogenesis in Vertebrates | 2.51 | 0.32 |
| PPARα/RXRα Activation | 2.51 | 0.28 |
| Role of Pattern Recognition Receptors in Recognition of Bacteria and Viruses | 2.51 | 0.30 |
| MSP-RON Signaling Pathway | 2.50 | 0.37 |
| IL-8 Signaling | 2.48 | 0.27 |
| Docosahexaenoic Acid (DHA) Signaling | 2.46 | 0.39 |
| Inhibition of Angiogenesis by TSP1 | 2.44 | 0.41 |
| Phospholipases | 2.37 | 0.35 |
| Molecular Mechanisms of Cancer | 2.36 | 0.25 |
| Wnt/Ca+ pathway | 2.35 | 0.35 |
| GADD45 Signaling | 2.35 | 0.47 |
| Altered T Cell and B Cell Signaling in Rheumatoid Arthritis | 2.33 | 0.32 |
| Role of Osteoblasts, Osteoclasts and Chondrocytes in Rheumatoid Arthritis | 2.32 | 0.26 |
| Circadian Rhythm Signaling | 2.30 | 0.39 |
| NAD biosynthesis II (from tryptophan) | 2.30 | 0.54 |
| Choline Biosynthesis III | 2.30 | 0.54 |
| Adipogenesis pathway | 2.25 | 0.29 |
| Role of IL-17F in Allergic Inflammatory Airway Diseases | 2.22 | 0.37 |
| Glucocorticoid Receptor Signaling | 2.22 | 0.25 |
| TR/RXR Activation | 2.19 | 0.31 |
| IL-9 Signaling | 2.17 | 0.38 |
| Xenobiotic Metabolism Signaling | 2.17 | 0.25 |
| Prolactin Signaling | 2.17 | 0.32 |
| STAT3 Pathway | 2.17 | 0.32 |
| Aldosterone Signaling in Epithelial Cells | 2.14 | 0.27 |
| Salvage Pathways of Pyrimidine Deoxyribonucleotides | 2.09 | 0.63 |
| IL-15 Signaling | 2.08 | 0.32 |
| Role of IL-17A in Arthritis | 2.08 | 0.33 |
| Triacylglycerol Degradation | 2.07 | 0.42 |
| eNOS Signaling | 2.06 | 0.28 |
| ERK/MAPK Signaling | 2.05 | 0.26 |
| Production of Nitric Oxide and Reactive Oxygen Species in Macrophages | 2.02 | 0.26 |
| iNOS Signaling | 2.01 | 0.35 |
| Hypoxia Signaling in the Cardiovascular System | 1.99 | 0.32 |
| p70S6K Signaling | 1.98 | 0.28 |
| Differential Regulation of Cytokine Production in Macrophages and T Helper Cells by IL-17A and IL-17F | 1.93 | 0.44 |
| Communication between Innate and Adaptive Immune Cells | 1.93 | 0.31 |
| Role of JAK family kinases in IL-6-type Cytokine Signaling | 1.93 | 0.40 |
| Macropinocytosis Signaling | 1.92 | 0.31 |
| VEGF Signaling | 1.92 | 0.29 |
| Regulation of the Epithelial-Mesenchymal Transition Pathway | 1.91 | 0.26 |
| Role of Cytokines in Mediating Communication between Immune Cells | 1.90 | 0.33 |
| UVB-Induced MAPK Signaling | 1.90 | 0.33 |
| RAR Activation | 1.89 | 0.26 |
| Protein Ubiquitination Pathway | 1.88 | 0.25 |
| Parkinson's Signaling | 1.88 | 0.47 |
| Glioma Signaling | 1.87 | 0.29 |
| Dendritic Cell Maturation | 1.86 | 0.26 |
| Superoxide Radicals Degradation | 1.86 | 0.67 |
| Growth Hormone Signaling | 1.85 | 0.30 |
| T Helper Cell Differentiation | 1.83 | 0.31 |
| Pathogenesis of Multiple Sclerosis | 1.81 | 0.56 |
| Nitric Oxide Signaling in the Cardiovascular System | 1.81 | 0.28 |
| Colorectal Cancer Metastasis Signaling | 1.80 | 0.25 |
| Antioxidant Action of Vitamin C | 1.79 | 0.29 |
| Death Receptor Signaling | 1.79 | 0.29 |
| VEGF Family Ligand-Receptor Interactions | 1.79 | 0.30 |
| Glioblastoma Multiforme Signaling | 1.77 | 0.26 |
| UVA-Induced MAPK Signaling | 1.77 | 0.29 |
| Type I Diabetes Mellitus Signaling | 1.77 | 0.28 |
| Role of NFAT in Regulation of the Immune Response | 1.75 | 0.26 |
| Eicosanoid Signaling | 1.74 | 0.31 |
| ILK Signaling | 1.74 | 0.25 |
| Graft-versus-Host Disease Signaling | 1.73 | 0.34 |
| FLT3 Signaling in Hematopoietic Progenitor Cells | 1.70 | 0.30 |
| Tec Kinase Signaling | 1.70 | 0.26 |
| Granzyme B Signaling | 1.70 | 0.44 |
| Putrescine Degradation III | 1.70 | 0.44 |
| Amyotrophic Lateral Sclerosis Signaling | 1.69 | 0.28 |
| Ovarian Cancer Signaling | 1.68 | 0.27 |
| Role of MAPK Signaling in the Pathogenesis of Influenza | 1.68 | 0.30 |
| IL-17A Signaling in Fibroblasts | 1.64 | 0.34 |
| Guanosine Nucleotides Degradation III | 1.64 | 0.46 |
| NAD Biosynthesis III | 1.63 | 0.75 |
| Neuregulin Signaling | 1.63 | 0.28 |
| IL-6 Signaling | 1.62 | 0.27 |
| NRF2-mediated Oxidative Stress Response | 1.61 | 0.25 |
| PDGF Signaling | 1.59 | 0.29 |
| Tumoricidal Function of Hepatic Natural Killer Cells | 1.59 | 0.38 |
| Estrogen-mediated S-phase Entry | 1.59 | 0.38 |
| Airway Pathology in Chronic Obstructive Pulmonary Disease | 1.56 | 0.57 |
| Tryptophan Degradation to 2-amino-3-carboxymuconate Semialdehyde | 1.56 | 0.57 |
| Ephrin A Signaling | 1.56 | 0.31 |
| TREM1 Signaling | 1.56 | 0.29 |
| Role of JAK2 in Hormone-like Cytokine Signaling | 1.55 | 0.34 |
| IL-12 Signaling and Production in Macrophages | 1.54 | 0.26 |
| IGF-1 Signaling | 1.50 | 0.27 |
| IL-17A Signaling in Gastric Cells | 1.47 | 0.36 |
| Reelin Signaling in Neurons | 1.47 | 0.28 |
| Sphingosine-1-phosphate Signaling | 1.45 | 0.26 |
| β-alanine Degradation I | 1.45 | 1.00 |
| Choline Degradation I | 1.45 | 1.00 |
| ErbB4 Signaling | 1.44 | 0.29 |
| IL-3 Signaling | 1.43 | 0.28 |
| CCR3 Signaling in Eosinophils | 1.42 | 0.26 |
| p53 Signaling | 1.40 | 0.27 |
| LPS/IL-1 Mediated Inhibition of RXR Function | 1.40 | 0.24 |
| Hereditary Breast Cancer Signaling | 1.38 | 0.26 |
| Oncostatin M Signaling | 1.36 | 0.32 |
| Retinoate Biosynthesis I | 1.36 | 0.33 |
| Retinol Biosynthesis | 1.36 | 0.33 |
| PTEN Signaling | 1.36 | 0.26 |
| Thrombopoietin Signaling | 1.35 | 0.29 |
| IL-10 Signaling | 1.35 | 0.28 |
| Gap Junction Signaling | 1.32 | 0.25 |
| Fatty Acid α-oxidation | 1.32 | 0.40 |
| Leptin Signaling in Obesity | 1.31 | 0.27 |
| HIF1α Signaling | 1.30 | 0.26 |
| Citrulline-Nitric Oxide Cycle | 1.30 | 0.60 |
